# The Genomics of Disulfide Bonding and Protein Stabilization in Thermophiles

**DOI:** 10.1371/journal.pbio.0030309

**Published:** 2005-08-23

**Authors:** Morgan Beeby, Brian D O'Connor, Carsten Ryttersgaard, Daniel R Boutz, L. Jeanne Perry, Todd O Yeates

**Affiliations:** **1** UCLA-DOE Institute for Genomics and Proteomics, University of California, Los Angeles, California, United States of America,; **2** Department of Chemistry and Biochemistry, University of California, Los Angeles, California, United States of America,; **3** Molecular Biology Institute, University of California, Los Angeles, California, United States of America; Brandeis UniversityUnited States of America

## Abstract

Thermophilic organisms flourish in varied high-temperature environmental niches that are deadly to other organisms. Recently, genomic evidence has implicated a critical role for disulfide bonds in the structural stabilization of intracellular proteins from certain of these organisms, contrary to the conventional view that structural disulfide bonds are exclusively extracellular. Here both computational and structural data are presented to explore the occurrence of disulfide bonds as a protein-stabilization method across many thermophilic prokaryotes. Based on computational studies, disulfide-bond richness is found to be widespread, with thermophiles containing the highest levels. Interestingly, only a distinct subset of thermophiles exhibit this property. A computational search for proteins matching this target phylogenetic profile singles out a specific protein, known as protein disulfide oxidoreductase, as a potential key player in thermophilic intracellular disulfide-bond formation. Finally, biochemical support in the form of a new crystal structure of a thermophilic protein with three disulfide bonds is presented together with a survey of known structures from the literature. Together, the results provide insight into biochemical specialization and the diversity of methods employed by organisms to stabilize their proteins in exotic environments. The findings also motivate continued efforts to sequence genomes from divergent organisms.

## Abstract 2

Certain thermophiles are found to stabilize their proteins in extreme environments with additional disulfide bonds. A phylogenetic profile identifies a protein disulfide oxidoreductase critical to the stabilization process.

## Introduction

Structural disulfide bonds are a covalent tertiary interaction in proteins, acting to stabilize a folded protein structure. Until recently, the classical view in biochemistry held that structural disulfide bonds are present almost exclusively in extracellular and compartmentalized proteins, as the reducing environment of the cytosol renders disulfide bonds only marginally stable [1,2]. In cellular compartments where disulfide bonding is abundant, such as the prokaryotic periplasm, disulfide-bond biochemistry is tightly regulated [3,4]. In the case of *Escherichia coli,* the DsbA–DsbB pathway in the periplasm, together with the thioredoxin and glutathione reductases in the cytoplasm, forms a cellular system that regulates disulfide-bond breakdown in the cytoplasm and formation in the periplasm. Interestingly, recent work has shown that alterations in these control mechanisms can make possible the formation of cytoplasmic disulfide bonds [5]. Indeed, it has been shown that certain mutants of *E. coli* can form protein disulfide bonds within the cytoplasm by utilizing thioredoxin as a disulfide exchange protein [6]. These studies illustrate how relatively small genetic changes can lead to cellular conditions that support intracellular protein disulfide formation in organisms with otherwise reducing cytosolic environments. The facility with which the cytosol of an ordinary bacterium can be manipulated to allow disulfide bonding relates to emerging revelations on disulfide bonding in unusual prokaryotes, particularly those of the thermophilic type.

Previous genomic studies by our laboratory provided computational and biochemical evidence for the idea that disulfide bonds in intracellular proteins are present in certain thermophiles (organisms of optimal growth temperature, *T*_opt_, above 50 °C) and hyperthermophiles (*T*_opt_ ≥ 80 °C) [7]. For the remainder of this paper, the term “thermophile” is used to refer to both thermophiles and hyperthermophiles. Here, multiple lines of computational and experimental evidence are presented that illustrate a widespread, yet nuanced, pattern of disulfide-bond utilization in intracellular proteins across 199 prokaryotes. The specific distribution of disulfides observed across these genomes suggests specialization in strategies used by organisms to stabilize their proteins. A comparative phylogenetic analysis is also described that provides compelling support for a specific protein, which has been named protein disulfide oxidoreductase (PDO) [8,9], in forming and maintaining intracellular disulfide bonds in thermophiles. A new crystal structure of another hyperthermophilic protein with three disulfide bonds is also presented along with a survey of disulfide bonding in known three-dimensional structures from thermophiles. We interpret these results as implying a widespread stabilizing role for these intracellular disulfide bonds in certain organisms.

These findings and other recent results call into question the long-held view that disulfide bonds must be rare in cytosolic proteins in all organisms. Some organisms have evidently modulated their internal biochemistry to enable disulfide bonding as a key mechanism for stabilizing their proteins at high temperatures.

## Results

### Computational Analysis of Disulfide Richness across Genomes

**Figure 1:**
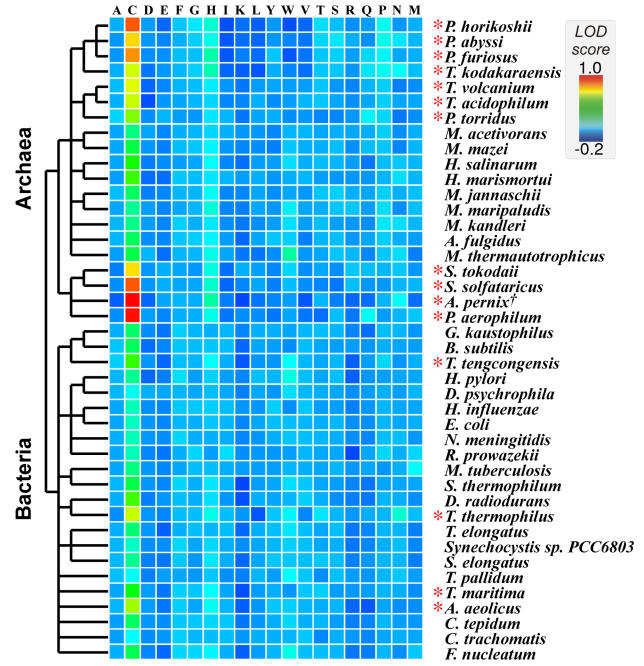
Predicted Protein Disulfide Abundance Across Thermophilic and Mesophilic Microorganisms For each genome, a colored row illustrates the tendency for cysteine residues in the proteins of that organism to occur close in three-dimensional space to each of the 20 amino acids. The amino acid types are given by their one-letter codes. The values reported are log (base 10) odds ratios, i.e., log ratio of observed over expected occurrences of proximal amino acids, with larger numbers implying a more frequent occurrence of amino acids in proximity. The figure illustrates only a subset of the sequenced organisms analyzed, but includes all the archaeal and bacterial thermophiles. The archaeal and bacterial major branches are noted and species names are provided. Some notable genomes with significant cysteine–cysteine proximity predictions include *P. aerophilum,* *A. pernix,* and *Py. furiosus.* Notably, cysteine–cysteine proximity stands out in thermophiles, particularly in the archaea, when compared with mesophiles such as *E. coli*. Furthermore, a red asterisk next to an organism name refers to the presence of the PDO protein (see text). Note that branch lengths are based on the National Center for Biotechnology Information taxonomy scheme and are not representative of phylogenetic distance, being used as a helpful visualization tool alone. An extended version featuring all genomes analyzed is available in the supporting online material (Figure S1). A dagger indicates that the value for *A. pernix* (1.236) exceeds the upper limit (1.0) of the coloring scheme used here.

A method for predicting from genomic data which organisms are rich in disulfide bonds has been described [7,10]. In the present study, a similar strategy was utilized in which genomic sequences are mapped onto the known three-dimensional structures of homologous proteins. Here, our analysis benefits from a vastly greater number of completely sequenced genomes. To begin, intracellular proteins were identified from the National Center for Biotechnology Information prokaryotic genome dataset (http://www.ncbi.nlm.nih.gov). If possible, each protein sequence was then matched to a known three-dimensional protein structure using either the BLAST or PSI-BLAST programs. The alignment of a query sequence to a homologous structure infers a likely three-dimensional mapping of the protein sequence in question, yielding homology-based structural predictions for many proteins. Considering all such protein sequences from a given genome as a group, the tendency of each amino acid type to appear in spatial proximity to every other type was then analyzed, taking into account the overall abundances of the 20 amino acid types. Enrichment in cysteine–cysteine proximity above the expected value was taken to indicate an enrichment of disulfide bonding. Since cysteine–cysteine proximity can also indicate metal-binding motifs, proteins were first filtered to remove proteins with metal-binding sites that would otherwise produce false-positive results. In addition, extracellular proteins in which structural disulfide bonds are expected to be observed were removed. These proximity criteria were also used to examine biases in pairwise amino acid proximity across all amino acid types beyond just cysteine–cysteine proximities (Figure 1).

Trends in pairwise amino acid proximities were measured for proteins from 199 distinct prokaryotic genomes, and close cysteine–cysteine pairings were interpreted as likely specific disulfide bonds in these organisms. While all possible pairings of amino acids were examined, close cysteine–cysteine proximity was, by far, the dominant trend in this investigation. With a few exceptions, thermophiles exhibited a pronounced bias in the spatial proximity of cysteine–cysteine residues, supporting a role for disulfide bonds in these organisms. Figure 1 illustrates this trend by showing the tendency of cysteine residues to be near all 20 types of amino acid in three dimensions for several organisms. Of all other possible pairwise combinations, tryptophan–tryptophan was the only other pairing observed to be significant according to our distance criteria, but in a smaller subset of organisms (data not shown). Previous work has established a role for aromatic clustering in thermophilic proteins, and our results may be an indication of this more subtle trend [11].

**Figure 2:**
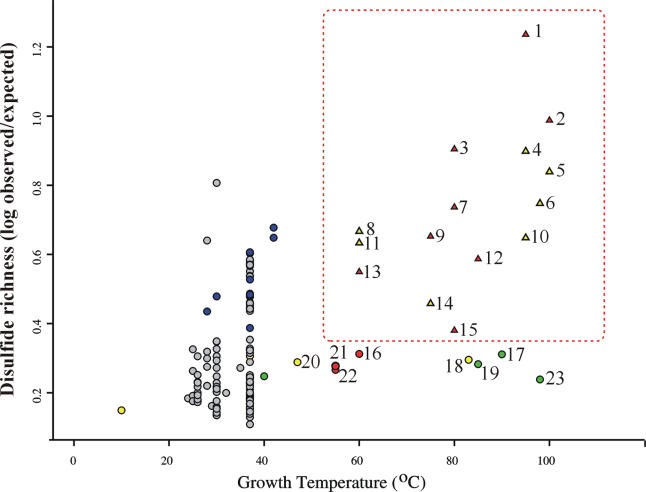
Correspondence of Growth Temperature and Disulfide Richness A plot of log ratios of cysteine–cysteine proximity versus optimal growth temperature for 99 sequenced genomes is presented. Optimal growth temperatures were taken from the German Collection of Microorganisms and Cell Cultures (DSMZ) and from genome sequence literature. Organisms are classified by color and symbol shape according to the following scheme: mesophiles that include annotations in the literature suggesting some extremophilic property other than thermophilicity (blue); mesophiles that do not include literature annotations suggesting extremophilic qualities (grey); sulfur-reducing bacteria and archaea (yellow); methanogenic bacteria and archaea (green); non-methanogenic/non-sulfur-reducing thermophiles (red); genomes that contain a PDO protein (triangle) (see text); and genomes that do not contain a PDO protein (circle). The genomes containing the PDO protein fit perfectly into the top right segment of the plot, as illustrated by the box drawn in dotted red lines. Numbers indicate the following organisms: 1, *A. pernix;* 2, *P. aerophilum;* 3, *S. solfataricus;* 4, *Py. horikoshii;* 5, *Py. furiosus;* 6, *Py. abyssi;* 7, *S. tokodaii;* 8, *Thermoplasma volcanium;* 9, *Thermus thermophilus* (both HB8 and HB27); 10, *Thermococcus kodakaraensis;* 11, *T. acidophilum;* 12, *Aquifex aeolicus;* 13, *Picrophilus torridus;* 14, *Thermoanaerobacter tengcongensis;* 15, *Thermotoga maritima;* 16, *Symbiobacterium thermophilum;* 17, *Methanothermobacter thermoautotrophicus;* 18, *Archaeoglobus fulgidus;* 19, *Methanococcus jannaschii;* 20, *Synechococcus elongatus;* 21, *Geobacillus kaustophilus;* 22, *Thermosynechococcus elongatus;* and 23, *Methanopyrus kandleri*.

The predicted disulfide abundance (expressed as a proximity score for cysteine–cysteine pairs) is shown in Figure 2 as a function of the maximum growth temperature of each organism. Disulfide richness is identified in thermophiles, both archaeal and bacterial. As expected, *Pyrobaculum aerophilum*, an organism singled out in earlier studies [7,12], exhibits a high propensity for cysteines to be in close proximity, with pairs of cysteine residues appearing in proximity nearly ten times more often than expected by chance. For *Aeropyrum pernix*, which shows the greatest enrichment in cysteine–cysteine proximity among all the organisms examined to date, cysteine proximity is higher by a factor of more than 17 times that which was expected on the basis of the total cysteine abundance in that organism. It is interesting to note that many of the organisms that appear to favor disulfide bonds have a reduced total cysteine abundance compared to other thermophiles and mesophiles [13]. This suggests the possibility of a significant evolutionary pressure against free (thiol) cysteines, and a concomitant elimination of cysteine residues lacking a structural (i.e., disulfide-bonded) or functional (i.e., metal-binding or catalytic) role in such organisms. Whether the placement of cysteines in the proteins of disulfide-rich organisms differs from the placement of cysteines in other organisms remains to be seen.

Interestingly, not all thermophilic organisms appear to contain an abundance of disulfides. Specifically, thermophiles with low disulfide richness include the methanogenic organisms and many of the sulfur-reducing organisms examined here, together with the few thermophilic cyanobacteria. Many of the thermophiles with low cysteine–cysteine pairwise proximity scores are strict anaerobes, growing at very low oxidation–reduction (redox) potentials (i.e., strongly reducing conditions). It may be that the environmental niche or the intrinsic biochemistry of these organisms precludes the significant use of cytosolic disulfide bonds.

In addition to thermophilic prokaryotes, certain other organisms appear to have measurably elevated degrees of disulfide bonding. These include some halophiles, alkalophiles, acidophiles, and radiation-tolerant organisms. This trend suggests that disulfide bonds might serve generally to stabilize proteins in a variety of extreme environments.

### Identification of a Candidate Protein Involved in Disulfide-Bond Formation in Thermophiles

The property of disulfide richness is distributed in a distinctive pattern across the phylogenetic tree, covering select thermophiles belonging to both the archaeal *and* bacterial domains of life. This suggests a phylogenetic approach for investigating the biochemical mechanisms related to disulfide maintenance. To investigate the hypothesis that proteins present exclusively in the most disulfide-rich thermophiles are involved in establishing or maintaining disulfide bonds, orthologous proteins that were present exclusively in these organisms were identified using techniques similar to ones developed previously [14]. Other studies aimed at identifying proteins involved in thermophilic adaptation [15,16] have been performed, but our study differs in certain respects. Earlier studies have operated under the implicit assumption that all thermophiles would use the same complement of proteins to survive at high temperatures. Here, we operate with the understanding that different organisms appear to use different mechanisms. In particular, the above analysis permits a focus on the disulfide-bonding mechanism. Thus we seek to identify protein(s) exclusive to the subset of organisms predicted here as having high levels of intracellular disulfide bonds.

**Figure 3:**
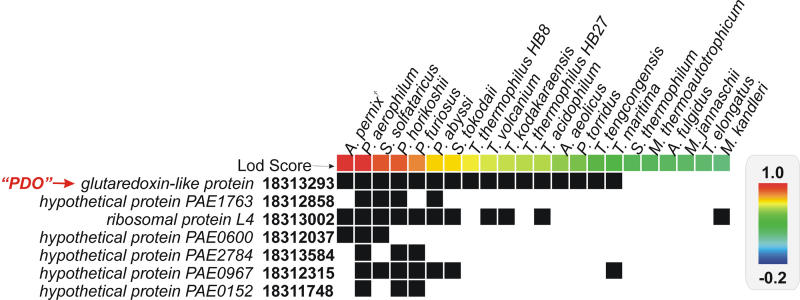
Identification of a Protein Exclusive to Disulfide-Rich Thermophiles Proteins were searched to find those exclusively present in organisms with high predicted abundance of protein disulfide bonds. Phylogenetic profiles are shown for the seven best protein matches according to our search criteria (see text). All thermophilic genomes are shown across the top, colored according to their predicted disulfide richness (see Figure 1). For each protein row (identified by its GI number), a black box indicates that the homologous protein is present in the genome represented in that column. A single protein, PDO (first profile, GI 18313293, previously annotated as a “glutaredoxin-like protein”, labeled here additionally as “PDO”), is singled out as being most closely correlated with disulfide richness and thermophilicity. Annotation here is taken directly from the annotation provided with the genome. PDO was previously annotated as a “glutaredoxin-like protein” based on its C-terminal similarity to glutaredoxin. A dagger indicates that the value for *A. pernix* (1.236) exceeds the upper limit (1.0) of the coloring scheme used here.

A small subset of proteins was identified as unique to these organisms (Figure 3). However, only one protein matched a template profile perfectly—a protein from a family previously described as containing possible PDOs [9]. This protein family was previously identified as exclusive to thermophiles, and its potential involvement in a subset of disulfide-rich organisms was noted [8]. Interestingly, proteins from this family were not detected in certain key organisms, notably *P. aerophilum*. Here, a more complete list of PDO proteins was found, and a strikingly precise correlation of the exclusive occurrence of PDO in thermophiles with high disulfide occurrence was discovered (see Figures 1 and 2). Intriguingly, the PDO family is not isolated to a single branch of the organismal tree (see Figure 1) and, as such, its precise co-occurrence with disulfide richness is particularly compelling evidence for a significant relationship to this special cellular property. Our findings therefore strongly reinforce the ideas of Pedone et al. [8] who have performed biochemical and structural characterization of the PDO protein from *Pyrococcus furiosus*.

### The Structure and Role of PDO in Disulfide-Rich Microbes

**Figure 4:**
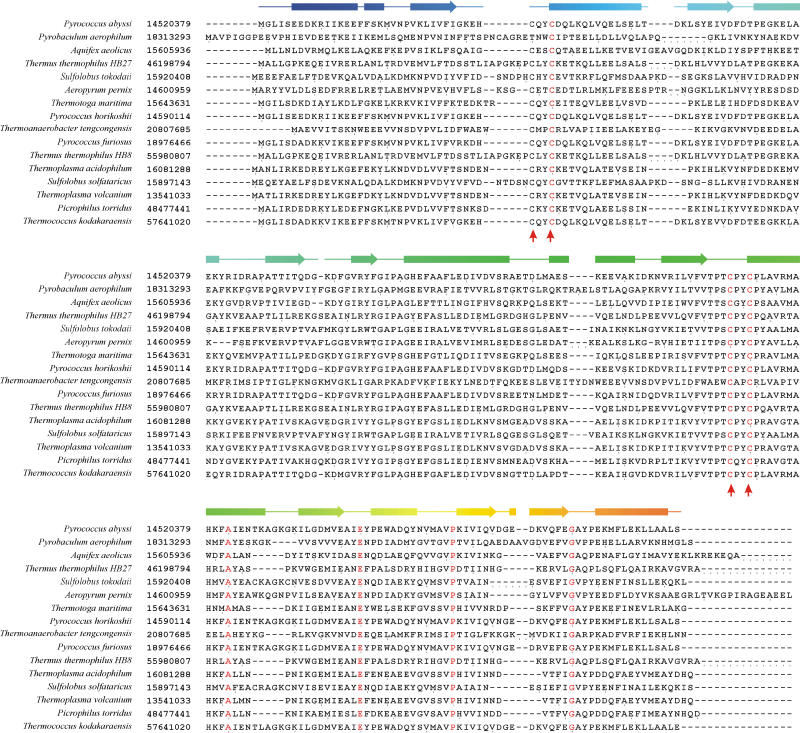
The PDO Family of Proteins A multiple sequence alignment of 16 members of the PDO family is shown. The two CxxC motifs are indicated by pairs of red arrows. Each protein is labeled with its organism of origin and GI number. Note the disrupted N-terminal CxxC motif in the *P. aerophilum* PDO sequence.

**Figure 5:**
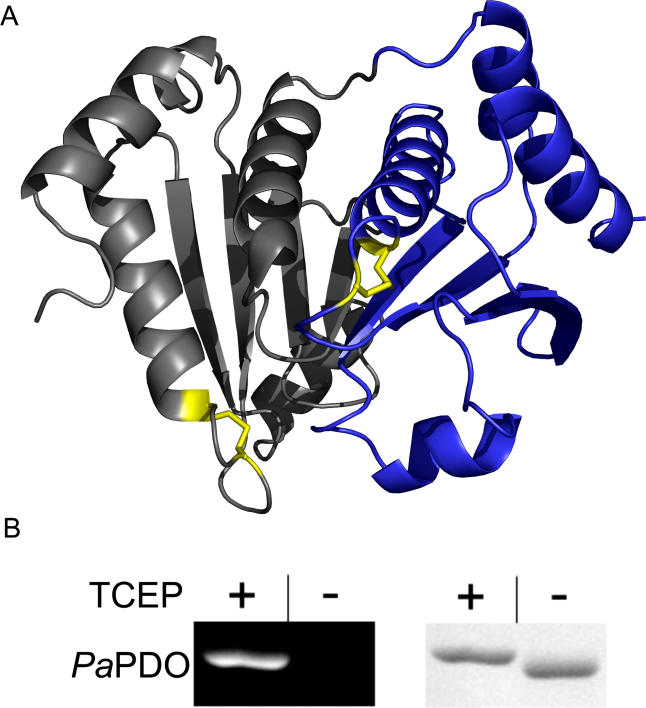
The Previously Determined PDO Structure and Evidence that the *P. aerophilum* Protein Has Similar Disulfide Bonding (A) The crystal structure of the PDO protein from *Py. furiosus* [12], PDB accession code 1A8L, showed that it contains two fused thioredoxin-like domains (colored grey and blue), with a single contiguous beta-sheet through both thioredoxin domains such that they effectively form one large domain. Each thioredoxin sub-domain bears a CxxC sequence motif with each pair of cysteines forming a disulfide bond (yellow), consistent with the prediction from the profile analysis that it could play a key role in intracellular protein disulfide-bond formation. Figure 5A was generated using PyMOL [54]. (B) Cysteines in purified recombinant *Pa*PDO exist predominantly in disulfide-bonded form. (Left gel) Denatured *Pa*PDO protein was reacted with the thiol-reactive reagent CPM to fluorescently label cysteines in the presence (+) or absence (−) of the strong reducing agent TCEP. Samples separated by sodium dodecyl sulfate–polyacrylamide gel electrophoresis clearly show a minimal labeling of the native protein (in the absence of reductant). (Right gel) Following fluorescence analysis, protein bands were stained with Coomassie Brilliant Blue to determine total protein present. The gel shows that reduced (+) and non-reduced (−) samples contained similar amounts of protein. The slightly lower position of the non-reduced *Pa*PDO compared to reduced *Pa*PDO is attributed to the presence of disulfide bonds in the non-reduced sample, which place constraints on the denatured state of the polypeptide and thus lead to a faster migration rate through the gel.

The PDO family, unique to disulfide-rich thermophiles, includes 16 known members from our set of fully sequenced genomes (Figure 4). The PDO protein from *Py. furiosus* has previously been structurally characterized [9] (Figure 5A). Its involvement in disulfide redox chemistry has already been established, where it has been shown to be capable of acting as a disulfide oxidase, reductase, or isomerase in vitro [8,17]. The crystal structure of *Py. furiosus* PDO shows two tandem domains of the thioredoxin/glutaredoxin-fold family. The C-terminal domain has clearly recognizable sequence similarity to glutaredoxins [9], explaining why PDO has not previously been detected in studies of thermophilic genome complements due to its homology-based classification as a member of the widely distributed glutaredoxin family. The observation of two thioredoxin folds in the PDO protein is provocative in view of the role that thioredoxin superfamily domains are known to play in disulfide-bond biochemistry, including reduction (e.g., thioredoxin), oxidation (e.g., DsbA), and isomerization (e.g., protein disulfide isomerase [PDI]) (reviewed in [18]).

Each of the two domains in PDO contains one CxxC sequence motif, with the exception of the *P. aerophilum* protein whose N-terminal CxxC motif is disrupted by an insert of five amino acids between the cysteines (see Figure 4). It was unclear from the sequence whether the *P. aerophilum* insert might disrupt the N-terminal redox site by preventing the cysteines from forming a disulfide bond. To determine whether this insertion affected the structure of the active site, the quantity of free thiols present in the protein was assayed. Purified recombinant PDO from *P. aerophilum* (*Pa*PDO) was reacted with the fluorescent thiol-reactive label 7-diethylamino-3-(4′-maleimidylphenyl)-4-methylcoumarin (CPM) under denaturing conditions in the presence or absence of the reductant tris(2-carboxyethyl)phosphine hydrochloride (TCEP). The denaturing conditions ensure that all cysteines are accessible to the modifying reagent. If the redox site was disrupted, the cysteines would not be able to form a disulfide bond in the native protein, and thus would exist as reactive free thiols. In fact, the native protein showed minimal labeling (∼8%) compared to the reduced and fully labeled control sample (Figure 5B), indicating that both redox sites exist predominantly in their oxidized, disulfide form in the native protein. These results suggest a potential functional relevance of this N-terminal segment, despite the insert observed in the *P. aerophilum* sequence.

Although the specific role the PDO protein might serve in the cell has not been elucidated fully [19], the results presented here suggest that it is involved in the formation or maintenance of intracellular protein disulfide bonds in disulfide-rich organisms, possibly by functioning as a cytoplasmic PDI. Based on its apparent cellular function as well as its tandem domain structure, a parallel can be drawn between PDO and the eukaryotic enzyme PDI, which also contains multiple tandem thioredoxin domains as noted by Freedman, et al. [19]. PDI resides in the endoplasmic reticulum where it catalyzes the isomerization of protein disulfide bonds in an oxidizing environment. It is possible to speculate that the enzyme used by eukaryotes to form protein disulfide bonds in the endoplasmic reticulum could have arisen from a similar enzyme in a disulfide-rich thermophile. Further studies will be required to test the predicted function of PDO, and to investigate its potential relationship to eukaryotic PDI, although the lack of a good genetic model organism in the thermophiles limits what can be done in vivo at the present time.

### Three Disulfide Bonds Revealed in the Structure of a Cysteine-Rich Protein from *P. aerophilum*

**Figure 6:**
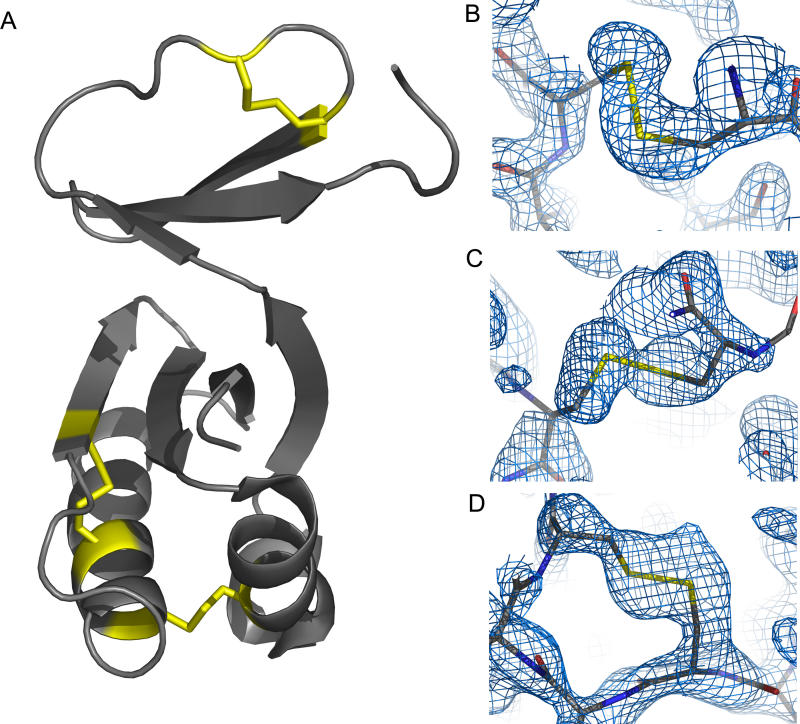
A Novel *P. aerophilum* Protein (A) A ribbon representation of the crystal structure of a novel *P. aerophilum* protein (GI 18312142) is presented. The six cysteine residues form three disulfide bonds (shown as stick models in yellow). (B–D) Simulated annealing electron density (“omit”) maps in the regions of the disulfide bonds between (B) Cys22 and Cys34, (C) Cys24 and Cys54, and (D) Cys80 and Cys83 are shown. All densities are contoured at 1 standard deviation. Images were generated using PyMOL [54].

Considering the apparent abundance of disulfide bonding in *P. aerophilum,* proteins containing multiple cysteines in their amino acid sequences would be expected to have a high likelihood of containing disulfide bonds. To test this, a 98-residue protein containing six cysteine residues was selected from the *P. aerophilum* genome [20] for structural characterization. The protein (GI 18312142) could not be assigned a function or three-dimensional fold in advance [20], as it had no recognizable sequence similarity to proteins of known function or structure. The crystal structure of the protein was determined to a resolution of 1.6 Å (Figure 6) with an R-factor of 18.4% (Table S1). The first 70 amino acid residues constitute an N-terminal domain whose three-dimensional fold has been observed previously in the copper chaperone Atx1 [21], but which does not contain the active-site residues of Atx1. The remaining 18 residues form a small C-terminal domain of novel fold that interacts with the N-terminal domain exclusively through hydrophobic contacts. The three-dimensional structure reveals that the six cysteine residues in the primary sequence are paired to form three disulfide bonds in the native fold (C22–C34, C24–C54, and C80–C83, Figure 6B). Although one disulfide bond (C80–C83) fits the sequence of a potential metal-binding/active-site CxxC motif [18], the C22–C34 and C24–C54 disulfide bonds do not fit any known metal-binding or active-site motifs and appear to serve structural roles within the protein fold. Of the 16 *P. aerophilum* proteins whose structures have been determined to date, a total of 29 cysteine residues have been visualized, and 23 of these have been found to form disulfide bonds. Despite the still relatively small sample size, these numbers provide important three-dimensional structural support for the claim of abundant disulfide bonds in this organism, meriting a further survey of thermophilic protein structures.

### Support of the Abundance of Cytosolic Disulfide Bonds in Thermophilic Organisms by Known Structures

Given the number of organisms predicted to have an abundance of cytosolic disulfide bonds, it would be expected that support for this would be evident in the structures of currently known proteins. Although the number of protein structures from thermophilic organisms is still low, trends are emerging that correspond to our predictions. A survey of the Protein Data Bank (http://www.rcsb.org/pdb) showed that 79 cytosolic proteins from thermophilic organisms exhibit at least one structural disulfide bond. Interestingly, organisms (with fully sequenced genomes) that are disulfide rich and encode PDO account for 71% of these disulfide-bonded structures. A survey of the structures available from the top four disulfide-rich organisms in our analysis (Table 1) revealed that 35.6% of the cysteines observed in these structures existed in the disulfide-bonded form. This stands in contrast to the case in *Bacillus subtilis* as a representative example, in which just 2.4% of the total number of cysteines in known structures formed disulfides. In every case where more than one cysteine is present within a *P. aerophilum* protein of known structure, a disulfide bond is found. Furthermore, *P. aerophilum* now accounts for three of the five known structures of thermophilic proteins containing three disulfide bonds (the most yet observed in a single cytosolic protein)—PDB IDs 1WY6, 1V4N, 1XQO, 1F1O, and 1RKI). Although the number of available structures is relatively low in this case, the prevalence of protein disulfide bonds in *P. aerophilum* proteins, as well as certain other organisms, stands in agreement with our predictions of disulfide abundance.

**Table 1:**
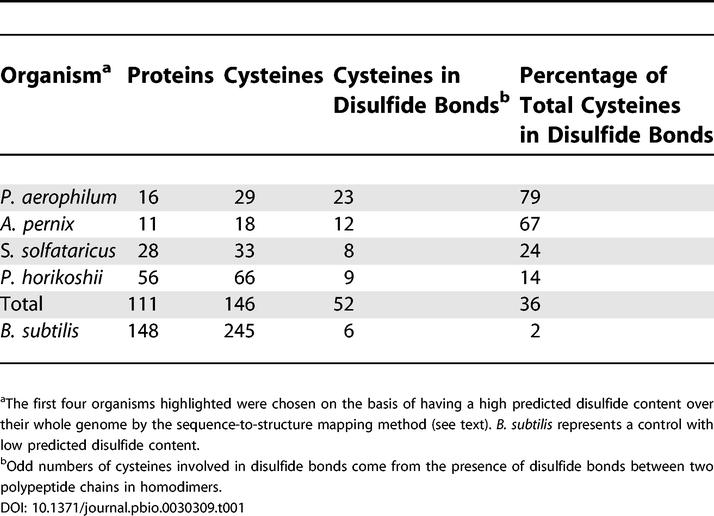
Summary of Disulfide-Bond Content in Protein Structures from Select Organisms

Disulfide bonds are a common occurrence in extracellular and compartmentalized proteins, where they are utilized to stabilize the folded proteins against the harsh conditions encountered there. The prevalence of disulfides in thermophilic proteins suggests that these bonds may serve a similar role to help stabilize proteins against thermal denaturation. Several stability studies of thermophilic proteins have provided evidence to support this role. Cacciapuoti et al. have shown that the 5′-methylthioadenosine phosphorylase from *Sulfolobus solfataricus* [22], as well as from *Py. furiosus* [23], contains stabilizing disulfide bonds. The 5′-methylthioadenosine phosphorylase from *Sulfolobus solfataricus* forms a homo-hexamer with three intermolecular disulfide bonds per complex, as confirmed by the crystal structure [24], while the homologous protein from *Py. furiosus* contains two intramolecular disulfide bonds [25]. Despite different disulfide patterns, both proteins exhibited a remarkable loss of activity upon exposure to the reducing agent dithiothreitol at optimal temperatures. A similar loss of activity occurred with the glycosyltrehalose trehalohydrolase from *S. solfataricus* upon mutational disruption of the intermolecular disulfide bond [26]. A decrease in melting temperature following disulfide disruption has been observed for *A. pernix* isocitrate dehydrogenase (ΔT_m_ = −9.6 °C) [27], *P. aerophilum* adenylosuccinate lyase (ΔT_m_ = −18.5 °C) [12], and *Py. woesei* TATA-binding protein (ΔT_m_ = −4 °C) [28]. Taken together, these results are indicative of a stabilizing role for certain disulfides in cytosolic proteins comparable to their well established structural role in extracellular and compartmentalized proteins. We are in the process of initiating a comprehensive proteomics study to identify proteins in *P. aerophilum* that contain inter- or intra-molecular disulfide bonds.

## Discussion

In this work, we describe a variety of computational and biochemical techniques used to imply the use of disulfide bonds as structural stabilization factors in some, but not all, thermophilic organisms. We also demonstrate the correlated presence of a specific protein, PDO, in those organisms thought to employ this mechanism. The discovery and analysis of disulfide-rich organisms provides an important illustration of how much remains to be learned about the diversity of life, as well as a clear example of the continued value of genomic data in exploring new biochemistry and cell biology. A role for disulfide bonds in the stabilization of intracellular thermophilic proteins has not been widely recognized, since—despite the concept's intuitive appeal—it seems to violate contemporary views of redox biochemistry. This study, together with a number of illustrative structures accumulated in recent years, means that the idea of structural disulfide bonds in cytoplasmic proteins in certain organisms must now be considered more routinely.

Numerous factors have previously been implicated in the stabilization of proteins in thermophiles. These include increased atomic packing, as suggested by the first hyperthermophilic enzyme structure determined by Chan et al. [29]; loop shortening, as shown on a genomic level by Thompson and Eisenberg [30]; and increased numbers of salt bridges as described by Karshikoff and Ladenstein [31]. The view that different proteins use disparate techniques for protein stabilization has been widely noted [32–37], and this study furthers the argument that there are multiple paths to protein stabilization.

The specific distribution of disulfide richness in a characteristic pattern across organisms is intriguing, presenting the possibility that different organisms may have evolved different solutions to the problem of protein stabilization. The disulfide-bond solution is particularly noteworthy in that it likely requires the presence of a specific protein, PDO. Disulfide bonding perhaps provides the most clearly delineated stabilization strategy thus far described, as a single covalent disulfide bond is able to effect a stabilization equivalent to that expected for numerous non-covalent stabilizing interactions acting together. It should be noted that not every protein in the organisms highlighted utilizes disulfide bonds for stability. Thus, we suggest that the methods employed in the stabilization of proteins, even from these thermophilic organisms, are a mosaic of all those mentioned above, with each organism employing different methods to varying degrees.

The discoveries presented here raise questions for further experimental studies. For example, what thermodynamic and kinetic considerations explain how certain organisms are able to use disulfide bonds for stability in the cytosol? Do these organisms have oxidizing cellular environments? Why have most mesophiles forgone the use of disulfide bonds for protein stabilization? We are currently in the process of identifying disulfide-bonded proteins in the lysate of certain thermophiles in order to shed light on questions like these. However, the lack of knowledge concerning the basic biology of many thermophiles, particularly the archaea, has limited the ability to investigate important aspects of this phenomenon. Research into the identification of the small-molecule thiols acting as redox buffer systems, as well as development of a genetic system for recombinant protein expression in these organisms, as is currently under way in the genus *Sulfolobus* (for review, see Ciaramella et al. [38]), will greatly enhance our ability to further investigate disulfide abundance.

With regard to the question of whether organisms rich in protein disulfide bonds must have oxidizing cellular environments, we hypothesize that regulation of disulfide-bond formation could be achieved through an interplay of thermodynamic and kinetic effects. The concept of a “reduction potential” for the entire cytoplasm is a synthesis of the reduction potentials of various molecular components of the cell. If thermodynamically favorable redox reactions are not kinetically aided by the presence of appropriate enzymes, the rates of those reactions could be so slow as to be effectively absent. Thus we argue that it may be possible to form deoxyribonucleotides (by reduction of ribonucleotides) for DNA synthesis using ribonucleotide reductase, while simultaneously allowing the formation of structural protein disulfide bonds in the cytoplasm, if these two pathways are kinetically separated. The non-equilibrium nature of cellular systems—enabled by enzymatic recognition and catalysis—makes it possible for two such seemingly opposing redox processes to coexist. We anticipate that the PDO protein may be only one facet of a complex disulfide-bond maintenance system.

The knowledge of disulfide richness in certain organisms suggests practical applications, including engineering enhanced protein stability and facilitating protein-fold recognition. Disulfide-rich organisms should allow the development of novel tools and approaches for attacking such problems of current interest. This work depends upon the availability of sequenced genomes, and the availability of additional thermophilic genomes has enabled the identification of an enigmatic protein family as a potential player in the biochemistry of cytoplasmic disulfide bonds. We hope this study will promote continued interest in sequencing more genomes from diverse organisms so as to further enhance the scope and resolution of comparative genomics techniques. As more genomes become available, we anticipate that the ease of discovery of specific genomic adaptations to the environment will improve and yield further insights into molecular evolution and cell biology.

## Materials and Methods

### 

#### Genomes

Predicted protein sequences from the National Center for Biotechnology Information (http://www.ncbi.nlm.nih.gov) for all genomes predicted to encode 700 or more proteins (199 prokaryotic genomes as of March 2005) were used for disulfide-bond predictions. Smaller genomes were discarded to safeguard against low signal-to-noise results. Pairwise amino acid proximity matrices (below) were calculated for each of these genomes.

#### Filtering

Extracellular proteins were removed using predictions from SignalP 2.0 (http://www.abcc.ncifcrf.gov/app/htdocs/appdb/index.php?info=protein) to detect signal peptides at the N-termini (first 70 residues), thus ensuring that the preponderance of proteins examined were intracellular [39]. A protein was considered to contain an export signal if at least one SignalP test was positive. Transmembrane proteins were identified and eliminated using TMPred (European Molecular Biology Laboratory, Heidelberg, Germany) [40] (version dated October 30, 1998) with a threshold value of 1,000 to remove proteins with potential extracellular or periplasmic domains. Proteins with known metal-binding motifs were also discarded to ensure that cysteines involved with metal binding were not included in the disulfide predictions. Motifs were identified from the Prosite database (Swiss-Prot Group, Swiss Institute of Bioinformatics, Geneva, Switzerland), and the ScanProsite 1.3 program (Swiss-Prot Group) [41] was used to exclude any proteins containing the motifs. Similarly, residues separated by fewer than four positions in the primary sequence were excluded as well as proteins with dual CxxC motifs. The end result was a dataset enriched for intracellular proteins, with cysteines not involved in metal-binding sites.

#### Pairwise amino acid proximity analysis

The process of mapping genomic protein sequences of unknown structure onto known structures was adapted from Mallick et al. [7]. Initially, 371,215 proteins from 199 prokaryotic organisms were queried against the Protein Data Bank (http://www.rcsb.org/pdb) [42] using BLAST (http://www.ncbi.nlm.nih.gov/Education/ BLASTinfo/information3.html) [43] (version dated April 23, 2002). If a hit was not identified with an *E-*value of <0.0001, the process was repeated with PSI-BLAST [44] (version dated April 23, 2002). When a homologous protein could be identified in the Protein Data Bank, the amino acid sequence of the query protein and the known structure were aligned with *mlocals*, an implementation of the Smith and Waterman local alignment algorithm from the Seqaln package (http://www-hto.usc.edu/software/seqaln) [45] (version 2.0). Based on this correspondence, three-dimensional coordinates were extracted for each amino acid position in the alignment. Those amino acids whose α-carbons were less than 8 Å apart and were separated by more than four positions in the primary sequence were tabulated by amino acid types. Predictions for disulfide bonds were made for specific proteins using these criteria. This criterion has previously been shown to predict disulfide bond state with ∼80% accuracy [7].

In addition, every pairing of amino acid types that met these criteria was examined. The number of times that particular pair was found in proximity was divided by the number expected by random chance, taking amino acid abundances into account. For display, these values were converted to the base 10 logarithm of the calculated odds ratios (LOD score). The resulting pairwise proximity score was used to measure biases in three-dimensional placement of all possible amino acid pairs. In the case of cysteine–cysteine pairs, the resulting pairwise proximity score was used as a general measure of disulfide richness for that organism. Specific disulfide predictions for proteins, and pairwise proximity matrices for all genomes examined, are available at http://www.doe-mbi.ucla.edu/Services/GDAP.

#### Identification of proteins exclusive to disulfide-rich organisms

The phylogenetic profile method [14] was used with some modification to search for proteins exclusively present in those genomes predicted to be disulfide rich. Orthologous protein families were defined using the BLAST program [43], where each *P. aerophilum* protein was used as a probe against the other 198 genomes. The process was then reversed with each protein from every genome queried with BLAST against the *P. aerophilum* genome to obtain a list of reciprocal best hits. These reciprocal best hits were further filtered such that probe-subject proteins were of roughly equivalent length. In this case, only those reciprocal best hits such that 0.9*L*_p_ ≤ *L*_s_ ≤ 1.1*L*_p_ were selected, where *L*_p_ is the length of the probe protein and *L*_s_ is the length of the subject protein. This resulted in a phylogenetic profile for each template protein in *P. aerophilum,* denoting patterns of presence and absence of orthologous proteins in the other organisms. This list was filtered for proteins exclusive to those thermophiles with high predicted levels of intracellular disulfide bonds by constructing a series of idealized template profiles by selecting the top *n* organisms as ranked by LOD score, for 6 ≤ *n* ≤ 23. Each of these templates was used to extract proteins with profiles that matched a template within a bit-distance of three. Multiple alignment of the PDO family was performed using ClustalW 1.82 (http://www.cbi.pku.edu.cn/Doc/tools/practices/evolution) [46] using the PAM alignment matrix with otherwise default parameters. Visualization was performed using SecSeq 1.0 [47] with secondary structure assignment based on the *Py. furiosus* PDO structure.

#### Experimental procedures

For the purposes of purification, crystallization, and structure determination, the protein (GI 18312142) was cloned into a pET-22b(+) expression vector, and expressed in *E. coli* BL21-Gold(DE3) (Novagen, Madison, Wisconsin, United States) as a histidine-tag fusion protein. Purification was carried out on a nickel column followed by removal of the histidine tag by thrombin cleavage and concentration to 36 mg/ml in 20 mM Tris–HCl (pH 8.0), 500 mM NaCl. Crystals were grown at 293 K by hanging-drop vapor diffusion, adding 1 μl of protein solution to 1 μl of well solution (0.1 M acetate [pH 4.6], 0.2 M Li_2_SO_4_, and 26% polyethylene glycol 8000). The crystals were transferred into a well solution containing an additional 20% (w/v) glycerol and then flash-frozen in liquid nitrogen. An in-house RU200 generator/R-Axis-IV detector (Rigaku, Tokyo, Japan) was used to collect X-ray diffraction data on a native crystal and two crystals soaked with potassium iodide and cesium chloride, respectively. The in-house native dataset was merged with a 1.6-Å native dataset collected at beamline 8.2.2. at Advanced Light Source (Berkeley, California, United States). All data were processed using Denzo and Scalepack (HKL Research, Charlottesville, Virginia, United States). The structure was solved by multiple isomorphous replacement using iodide and cesium sites located by SHELXD (http://shelx.uni-ac.gwdg.de/SHELX) [48]. The heavy-atom coordinates were refined with MLPHARE followed by solvent flattening using DM, both in the CCP4 [49] suite of programs (Collaborative Computational Project; http://www.ccp4.ac.uk). A traceable electron-density map was subsequently produced and a model was built using the program O [50] (http://xray.bmc.uu.se/~alwyn/Distribution/distrib_frameset.html). Initial rounds of refinement were performed using simulated annealing as implemented in CNS (http://cns.csb.yale.edu/v1.1) [51], and later steps of the refinement were carried out with REFMAC5 in CCP4. The model contains two chains with residues 1–101 and 1–97, respectively, as well as two fragments of polyethylene glycol—hexaethylene glycol and tetraethylene glycol, together with two sulfate ions and one chloride ion. The quality of the model was evaluated with the ERRAT (http://www.doe-mbi.ucla.edu/Services/ERRAT) [52] and PROCHECK (http://www.biochem.ucl.ac.uk/bsm/biocomp) [53] programs. Details of the data collections and refinement are shown in Table S1.

To enable the determination of the redox state of *Pa*PDO cysteines, the protein (GI 18313293) was cloned into a pET-16b expression vector and expressed in *E. coli* as a histidine-tag fusion protein. Cells were lysed by sonication in lysis buffer (50 mM Tris [pH 8.0], 0.2% NP40, 300 mM NaCl, and 10% glycerol) and centrifuged. The supernatant was collected and an initial heat-purification step was performed by heating at 80–85 °C in a water bath, denaturing the majority of the *E. coli* proteins. The supernatant was then passed over a nickel column and the protein eluted using an imidazole gradient. Finally, the eluant was run on a gel filtration column and the fractions corresponding to PDO pooled. Purified recombinant *Pa*PDO was diluted to 0.1 mg/ml in denaturation buffer (1% SDS, 10 mM Tris [pH 8.0], and 10 mM EDTA) and divided into non-reduced and reduced samples. Both samples were heated to 95 °C for 3 min to denature them. For the non-reduced sample, a 5-fold excess of CPM (Molecular Probes, Eugene, Oregon, United States) was added prior to heating to ensure immediate labeling of exposed thiols. Following heat denaturation, the reduced sample was reacted with 10 mM TCEP (Sigma, St. Louis, Missouri, United States) for 20 min at room temperature to reduce disulfide-bonded cysteines. Following the reduction reaction, both non-reduced and reduced samples were reacted with a 10-fold excess of CPM in the dark at room temperature for 20 min. Samples were mixed with 2× SDS-PAGE sample loading buffer and run on a 12% acrylamide gel. Gels were imaged on AlphaImager 2200 (Alpha Innotech, San Leandro, California, United States). In-gel fluorescence was quantified using AlphaEase 5.5 (Alpha Innotech). Fluorescence of the non-reduced sample was below reasonable detection at the point of signal saturation for the reduced sample, so a series of 2-fold dilutions of the reduced sample was carried out to compare more accurately the amount of labeling of non-reduced relative to reduced sample. Following fluorescence analysis, gels were stained with Coomassie Brilliant Blue (Sigma) and imaged on AlphaImager 2200 (Alpha Innotech).

## Supporting Information

Figure S1 Trends in Apparent Disulfide Abundance across Thermophilic and Mesophilic Microorganisms For each genome, a colored row illustrates the tendency for cysteine residues in the encoded proteins to occur in spatial proximity to each of the 20 types of amino acids, including cysteine itself. The amino acid types are given by their one-letter codes (C = cysteine). The values reported are log (base 10) odds ratios. The archaeal and bacterial major branches are noted and organism names are provided. Some notable genomes include *P. aerophilum,* *A. pernix,* and *E. coli*. Cysteine–cysteine proximity stands out in thermophiles, particularly in the archaea, when compared with mesophiles such as *E. coli*. An asterisk indicates that the value for *A. pernix* (1.236) exceeds the upper limit (1.0) of the coloring scheme used here.
(26.6 MB TIF).

Table S1 Statistics of Data Reduction and Refinement (22 KB DOC).

### Accession Numbers

The GenBank (http://www.ncbi.nlm.nih.gov/Genbank) accession number for *Py. furiosus* is 18976466, and the Protein Data Bank (http://www.rcsb.org/pdb) accession number for the *Py. furiosus* PDO structure is 1A8L. The GenBank accession number for the 98-residue protein containing six cysteine residues selected from the *P. aerophilum* genome is 18312142. Atomic coordinates for protein GI 18312142 have been deposited in the Protein Data Bank under accession code 1RKI.

## Abbreviations

CPM: 7-diethylamino-3-(4′-maleimidylphenyl)-4-methylcoumarin
PaPDO: *Pyrobaculum aerophilum* PDO
PDI: protein disulfide isomerase
PDO: protein disulfide oxidoreductase
redox: oxidation–reduction
TCEP: tris(2-carboxyethyl)phosphine hydrochloride

## References

[pbio-0030309-b01] Branden C, Tooze J (1991). Introduction to protein structure.

[pbio-0030309-b02] Fahey RC, Hunt JS, Windham GC (1977). On the cysteine and cystine content of proteins. Differences between intracellular and extracellular proteins. J Mol Evol.

[pbio-0030309-b03] Hiniker A, Bardwell JC (2003). Disulfide bond isomerization in prokaryotes. Biochemistry.

[pbio-0030309-b04] Kadokura H, Katzen F, Beckwith J (2003). Protein disulfide bond formation in prokaryotes. Annu Rev Biochem.

[pbio-0030309-b05] Bessette PH, Aslund F, Beckwith J, Georgiou G (1999). Efficient folding of proteins with multiple disulfide bonds in the *Escherichia coli* cytoplasm. Proc Natl Acad Sci U S A.

[pbio-0030309-b06] Masip L, Pan JL, Haldar S, Penner-Hahn JE, DeLisa MP (2004). An engineered pathway for the formation of protein disulfide bonds. Science.

[pbio-0030309-b07] Mallick P, Boutz DR, Eisenberg D, Yeates TO (2002). Genomic evidence that the intracellular proteins of archaeal microbes contain disulfide bonds. Proc Natl Acad Sci U S A.

[pbio-0030309-b08] Pedone E, Ren B, Ladenstein R, Rossi M, Bartolucci S (2004). Functional properties of the protein disulfide oxidoreductase from the archaeon *Pyrococcus furiosus*: A member of a novel protein family related to protein disulfide-isomerase. Eur J Biochem.

[pbio-0030309-b09] Ren B, Tibbelin G, de Pascale D, Rossi M, Bartolucci S (1998). A protein disulfide oxidoreductase from the archaeon *Pyrococcus furiosus* contains two thioredoxin fold units. Nat Struct Biol.

[pbio-0030309-b10] O'Connor BD, Yeates TO (2004). GDAP: A web tool for genome-wide protein disulfide bond prediction. Nucleic Acids Res.

[pbio-0030309-b11] Kannan N, Vishveshwara S (2000). Aromatic clusters: A determinant of thermal stability of thermophilic proteins. Protein Eng.

[pbio-0030309-b12] Toth EA, Worby C, Dixon JE, Goedken ER, Marqusee S (2000). The crystal structure of adenylosuccinate lyase from *Pyrobaculum aerophilum* reveals an intracellular protein with three disulfide bonds. J Mol Biol.

[pbio-0030309-b13] Rosato V, Pucello N, Giuliano G (2002). Evidence for cysteine clustering in thermophilic proteomes. Trends Genet.

[pbio-0030309-b14] Pellegrini M, Marcotte EM, Thompson MJ, Eisenberg D, Yeates TO (1999). Assigning protein functions by comparitive genome analysis: Protein phylogenetic profiles. Proc Natl Acad Sci U S A.

[pbio-0030309-b15] Forterre P (2002). A hot story from comparative genomics: Reverse gyrase is the only hyperthermophile-specific protein. Trends Genet.

[pbio-0030309-b16] Makarova KS, Wolf YI, Koonin EV (2003). Potential genomic determinants of hyperthermophily. Trends Genet.

[pbio-0030309-b17] Bartolucci S, de Pascale D, Rossi M (2001). Protein disulfide oxidoreductase from *Pyrococcus furiosus* Biochemical properties. Methods Enzymol.

[pbio-0030309-b18] Fomenko DE, Gladyshev VN (2003). Identity and functions of CxxC-derived motifs. Biochemistry.

[pbio-0030309-b19] Freedman RB (1998). Novel disulfide oxidoreductase in search of a function. Nat Struct Biol.

[pbio-0030309-b20] Fitz-Gibbon ST, Ladner H, Kim UJ, Stetter KO, Simon MI (2002). Genome sequence of the hyperthermophilic crenarchaeon *Pyrobaculum aerophilum*. Proc Natl Acad Sci U S A.

[pbio-0030309-b21] Rosenzweig AC, Huffman DL, Hou MY, Wernimont AK, Pufahl RA (1999). Crystal structure of the Atx1 metallochaperone protein at 1.02 Å resolution. Structure Fold Des.

[pbio-0030309-b22] Cacciapuoti G, Porcelli M, Bertoldo C, De Rosa M, Zappia V (1994). Purification and characterization of extremely thermophilic and thermostable 5′-methylthioadenosine phosphorylase from the archaeon *Sulfolobus solfataricus*. Purine nucleoside phosphorylase activity and evidence for intersubunit disulfide bonds. J Biol Chem.

[pbio-0030309-b23] Cacciapuoti G, Bertoldo C, Brio A, Zappia V, Porcelli M (2003). Purification and characterization of 5′-methylthioadenosine phosphorylase from the hyperthermophilic archaeon *Pyrococcus furiosus* Substrate specificity and primary structure analysis. Extremophiles.

[pbio-0030309-b24] Appleby TC, Mathews II, Porcelli M, Cacciapuoti G, Ealick SE (2001). Three-dimensional structure of a hyperthermophilic 5′-deoxy-5′-methylthioadenosine phosphorylase from *Sulfolobus solfataricus*. J Biol Chem.

[pbio-0030309-b25] Cacciapuoti G, Moretti MA, Forte S, Brio A, Camardella L (2004). Methylthioadenosine phosphorylase from the archaeon *Pyrococcus furiosus*. Mechanism of the reaction and assignment of disulfide bonds. Eur J Biochem.

[pbio-0030309-b26] Feese MD, Kato Y, Tamada T, Kato M, Komeda T (2000). Crystal structure of glycosyltrehalose trehalohydrolase from the hyperthermophilic archaeum *Sulfolobus solfataricus*. J Mol Biol.

[pbio-0030309-b27] Karlstrom M, Stokke R, Steen IH, Birkeland NK, Ladenstein R (2005). Isocitrate dehydrogenase from the hyperthermophile *Aeropyrum pernix* X-ray structure analysis of a ternary enzyme-substrate complex and thermal stability. J Mol Biol.

[pbio-0030309-b28] DeDecker BS, O'Brien R, Fleming PJ, Geiger JH, Jackson SP (1996). The crystal structure of a hyperthermophilic archaeal
TATA-box binding protein. J Mol Biol.

[pbio-0030309-b29] Chan MK, Mukund S, Kletzin A, Adams MW, Rees DC (1995). Structure of a hyperthermophilic tungstopterin enzyme, aldehyde ferredoxin oxidoreductase. Science.

[pbio-0030309-b30] Thompson MJ, Eisenberg D (1999). Transproteomic evidence of a loop-deletion mechanism for enhancing protein thermostability. J Mol Biol.

[pbio-0030309-b31] Karshikoff A, Ladenstein R (2001). Ion pairs and the thermotolerance of proteins from hyperthermophiles: A “traffic rule” for hot roads. Trends Biochem Sci.

[pbio-0030309-b32] Chakravarty S, Varadarajan R (2002). Elucidation of factors responsible for enhanced thermal stability of proteins: A structural genomics based study. Biochemistry.

[pbio-0030309-b33] Kumar S, Nussinov R (2001). How do thermophilic proteins deal with heat?. Cell Mol Life Sci.

[pbio-0030309-b34] Vieille C, Zeikus GJ (2001). Hyperthermophilic enzymes: Sources, uses, and molecular mechanisms for thermostability. Microbiol Mol Biol Rev.

[pbio-0030309-b35] Jaenicke R, Bohm G (1998). The stability of proteins in extreme environments. Curr Opin Struct Biol.

[pbio-0030309-b36] Petsko GA (2001). Structural basis of thermostability in hyperthermophilic proteins, or “there's more than one way to skin a cat”. Methods Enzymol.

[pbio-0030309-b37] Rees DC, Adams MW (1995). Hyperthermophiles: Taking the heat and loving it. Structure.

[pbio-0030309-b38] Ciaramella M, Pisani FM, Rossi M (2002). Molecular biology of extremophiles: Recent progress on the hyperthermophilic archaeon *Sulfolobus*. Antonie Van Leeuwenhoek.

[pbio-0030309-b39] Nielsen H, Engelbrecht J, Brunak S, von Heijne G (1997). Identification of prokaryotic and eukaryotic signal peptides and prediction of their cleavage sites. Protein Eng.

[pbio-0030309-b40] Hofmann K, Stoffel W (1993). TMBASE—A database of membrane spanning protein segments. Biol Chem Hoppe-Seyler.

[pbio-0030309-b41] Gattiker A, Gasteiger E, Bairoch A (2002). ScanProsite: A reference implementation of a PROSITE scanning tool. Appl Bioinformatics.

[pbio-0030309-b42] Berman HM, Battistuz T, Bhat TN, Bluhm WF, Bourne PE (2002). The Protein Data Bank. Acta Crystallogr D Biol Crystallogr.

[pbio-0030309-b43] Altschul SF, Gish W, Miller W, Myers EW, Lipman DJ (1990). Basic local alignment search tool. J Mol Biol.

[pbio-0030309-b44] Altschul SF, Madden TL, Schaffer AA, Zhang J, Zhang Z (1997). Gapped BLAST and PSI-BLAST: A new generation of protein database search programs. Nucleic Acids Res.

[pbio-0030309-b45] Smith TF, Waterman MS (1981). Identification of common molecular subsequences. J Mol Biol.

[pbio-0030309-b46] Thompson JD, Higgins DG, Gibson TJ (1994). CLUSTAL W: Improving the sensitivity of progressive multiple sequence alignment through sequence weighting, position-specific gap penalties and weight matrix choice. Nucleic Acids Res.

[pbio-0030309-b47] Brodersen DE (2005). SecSeq, version 1.0 [computer program].

[pbio-0030309-b48] Uson I, Sheldrick GM (1999). Advances in direct methods for protein crystallography. Curr Opin Struct Biol.

[pbio-0030309-b49] Collaborative Computational Project, Number 4 (1994). The CCP4 suite: Programs for protein crystallography. Acta Crystallogr D Biol Crystallogr.

[pbio-0030309-b50] Jones TA, Zou JY, Cowan SW, Kjeldgaard (1991). Improved methods for building protein models in electron density maps and the location of errors in these models. Acta Crystallogr A.

[pbio-0030309-b51] Brunger AT, Adams PD, Clore GM, DeLano WL, Gros P (1998). Crystallography & NMR system: A new software suite for macromolecular structure determination. Acta Crystallogr D Biol Crystallogr.

[pbio-0030309-b52] Colovos C, Yeates TO (1993). Verification of protein structures: Patterns of nonbonded atomic interactions. Protein Sci.

[pbio-0030309-b53] Laskowski R, MacArthur M, Moss D, Thornton J (1993). PROCHECK: A program to check the stereochemical quality of protein structures. J Appl Cryst.

[pbio-0030309-b54] DeLano WL (2002). The PyMOL Molecular Graphics System.

