# Supplementary material for: The Genomics of Disulfide Bonding and Protein Stabilization in Thermophiles
Source: PLoS Biol. 2005 Aug 23;3(9):e309. doi: 10.1371/journal.pbio.0030309 (PMC1188242; doi:10.1371/journal.pbio.0030309)
Supplement: Supplementary file 1 [file pbio.0030309.st001.doc]

**Supplementary Table 1. Statistics of data reduction and refinement.** Atomic coordinates have been deposited in the Protein Data Bank under accession code 1RKI.

| **Data collection** | | | | | | | |
| --- | --- | --- | --- | --- | --- | --- | --- |
| Space group | C2 | | | | | | |
| Data set | *Native 1* | | *NaI* | | *CsCl* | *Native 2* | *Native-merged* |
| Unit cell |  | |  | |  |  |  |
| a (Å) | 104.9 | | 105.2 | | 104.9 | 105.5 | 105.5 |
| b (Å) | 43.6 | | 44.3 | | 44.2 | 43.7 | 43.7 |
| c (Å) | 52.5 | | 53.0 | | 52.8 | 52.7 | 52.7 |
| β (°) | 113.2 | | 113.9 | | 113.8 | 113.2 | 113.2 |
| Wavelength (Å) | 1.5418 | 1.5418 | | 1.5418 | | 1.1271 |  |
| Resolution (Å) | 80-2.0 | | 90-2.0 | | 90-2.1 | 90-1.6 | 90-1.6 |
| Total observations | 77265 | | 107034 | | 92601 | 138846 | 188616 |
| Unique reflections | 14631 | | 15239 | | 13027 | 27079 | 28068 |
| Completeness (%) | 99.0 (96.6) ^1^ | | 99.6 (98.4) ^1^ | | 98.5 (96.4) ^1^ | 92.6 (75.4) ^1^ | 96.0 (75.0) ^1^ |
| R_merge_^2^ (%) | 8.5 (30.7) ^1^ | | 11.5 (53.4) ^1^ | | 10.2 (33.4) ^1^ | 10.7 (52.9) ^1^ | 12.5 (49.5) ^1^ |
| Heavy atom sites | N/A | | 10 | | 7 | N/A | N/A |

| **Refinement** | |
| --- | --- |
| Protein atoms | 1623 |
| Ligand atoms^3^ | 47 |
| Solvent atoms | 89 |
| R_work_ (R_free_) (%)^4^ | 18.4 (23.5) |
| Average B-factor (Å^2^) | 30.7 |
| Deviation from ideal geometry |  |
| Bonds (Å) | 0.024 |
| Angles (°) | 2.27 |
| Ramachandran plot |  |
| Most favored regions (%) | 92.7 |
| Additionally allowed regions (%) | 7.3 |
| Generously allowed regions (%) | 0 |
| Disallowed regions (%) | 0 |

^1^Highest resolution shell.

^2^ R_merge_ = ∑_h_∑_i_ |I_hi_-<I_h_>|/∑<I_h_>, where I_hi_ is the intensity of the i^th^ observation of reflection h, and <I_h_> is the average intensity of redundant measurements of the h reflections.

^3^Hexaethyleneglycol, tetraethyleneglycol, acetic acid, chloride and two sulfate ions.

^4^R_work_ = ∑||F_o_|-|F_c_||/∑|F_o_|, where F_o_ and F_c_ are the observed and calculated structure-factor amplitudes. R_free_ is monitored with 1409 reflections excluded from refinement.
